# An item response theory analysis of the Dissociative Experiences Scale II: examining psychometric properties and longitudinal stability among Japanese adults

**DOI:** 10.1186/s12888-024-06465-w

**Published:** 2025-01-09

**Authors:** Tatsuya Ikeda, Yuhei Urano

**Affiliations:** 1 Graduate School of Education, Hyogo University of Teacher Education, Kobe, Japan; 2 Faculty of Education, Art and Science, Yamagata University, Yamagata, Japan

**Keywords:** Dissociative Experiences Scale II, Item response theory, Longitudinal stability, Psychometric properties

## Abstract

**Background:**

The Dissociative Experiences Scale (DES-II) is widely used globally. However, psychometric properties of the scale have not been adequately examined. The present study aimed to examine the psychometric properties and longitudinal stability of the DES-II.

**Method:**

We collected data at two time points, approximately three and a half years apart. At Time 1 (T1), 1029 participants (515 females, 514 males) with a mean age of 44.64 (± 14.02) responded to the survey. Out of the T1 participants, 210 individuals (105 females, 105 males) also responded to the T2 survey. We conducted item parameters of the DES-II with item response theory (IRT).

**Results:**

Our results showed that the DES-II is suitable for measuring strong dissociative traits, with all items displaying high discriminative power. The cut-off points for the DES-II were within a good range of measurement accuracy, and longitudinal stability over approximately three and a half years was adequate.

**Conclusion:**

In the present study, we applied item response theory (IRT) to the DES-II, which has traditionally been interpreted using classical test theory (CTT). Results suggested the need for item-focused assessment rather than relying solely on mean scores or cut-off points. Specifically, results suggested that the severity levels differed across item ratings, and to set cut-off points for each item based on the severity of the ratings. Furthermore, the possibility of cultural differences in response patterns of the DES-II was indicated. However, few studies have discussed cultural differences based on IRT; hence, further research should examine response patterns of the DES-II across various cultures. In conclusion, the DES-II is a valuable tool for assessing dissociative symptoms, with adequate psychometric properties from an item response theory perspective. Clinicians should consider item-specific responses in their assessments, and further research is needed to explore the scale's applicability across diverse populations.

## Introduction

Dissociation, defined as "disruption or discontinuity in usually integrated psychological function such as memory, consciousness, perception, sense of self and agency, or sensorimotor ability" [1], is observed in approximately 10% [2, 3] to 29% [4] of medical settings. According to a meta-analysis [5], numerous mental disorders may accompany dissociative symptoms. Furthermore, dissociation is known to coexist with mental disorders other than dissociative disorders, making them more severe [6, 7]. Considering that dissociation increases the risk of self-destructive behaviors such as suicide and self-harm [8], it is crucial to develop a well-validated tool to accurately assess dissociative symptoms.

There are various ways to assess dissociation: assessment methods for dissociation include interview-based and questionnaire-based approaches. The former includes the Dissociative Disorder Interview Schedule (DDIS) [9] and the Structured Clinical Interview for DSM-IV Dissociative Disorders-Revised (SCID-D-R) [10] based on the Diagnostic and Statistical Manual of Mental Disorders (DSM). The latter includes the Dissociative Experience Scale (DES) [11] and the Multidimensional Inventory of Dissociation (MID) [12]. Among these, interview-based methods require training and proficiency and are time-consuming to administer. On the other hand, questionnaire-based methods can be administered more quickly and conveniently than interview-based methods; Frankel [13] recommends the use of the DES.

Individuals with strong dissociative traits are observed not only in medical settings but also elsewhere. A community sample survey by Akyüz et al. [14] found that 1.7% of participants were diagnosed with some form of dissociative disorder, with 47.05% of them having dissociative identity disorder. Şar et al. [15] reported a disability-adjusted prevalence rate of dissociative disorders in women of 18.3% based on a survey of community samples. These studies both suggest that dissociative symptoms and dissociative disorders may exist undiagnosed and untreated in the general population. Considering the prevalence of potential dissociative disorders in the general population, the DES may be useful in screening individuals outside of medical settings.

Considering that the DES is a widely used scale for assessing dissociation, a detailed examination of the scale will enable comprehensive assessment. The DES [11] is a self-report measure consisting of 28 items. There are two response formats: one using a Visual Analogue Scale [11], referred to as the DES-I, and another using an 11-point Likert scale [16], referred to as the DES-II. The DES have been translated into various languages across cultures, with the reliability and validity confirmed for each (e.g. [16–19]). As mentioned later, cut-off points are established for the DES; in screening and assessing dissociation, both the DES scores and cut-off points are referenced. Hence, for accurate assessment, it is necessary to thoroughly examine the psychometric properties of the DES.

Screening of dissociation using the DES involves the use of cut-off points and Taxon item scores. Previous studies have set cut-off points at 15–20 [20], 30 [21, 22], and 45–55 [23], classifying individuals surpassing these scores as having high dissociation or subclinical dissociation. Additionally, a previous study [24] identified eight Taxon items which are DES-II items reflected pathological dissociative experiences. According to Waller et al. [24], the higher the score on the Taxon or the Taxon mean score (DES-Taxon (DES-T) score), the more pathological dissociation observed. Apart from the Taxon items, the remaining 20 items constitute the Normal Dissociation Index (NDI) [24].

The DES-II is a scale with excellent reliability. Internal consistency (i.e., Cronbach’s *α*) in previous studies ranged from 0.93 to 0.96 [17, 19, 25, 26], stability (i.e., intraclass correlation coefficients: ICC) ranged from 0.80 to 0.93 [23, 27], and test–retest reliability (i.e., correlation coefficients) ranged from 0.93 to 0.94 [19, 25]. Due to its high stability, dissociation measured by the DES-II is often considered as a personal trait [26, 28, 29].

However, the DES-II faces two limitations: (a) lack of thorough examination of psychometric properties, and (b) lack of examination regarding longitudinal stability. Firstly, there is insufficient evidence regarding the psychometric properties of the DES-II. Although Waller et al. [24] identified Taxon items through taxometric analysis, studies examining the factor structure of the DES through factor analysis (e.g., [30–32]) did not extract factors composed of Taxon items. Therefore, it is unclear whether Taxon-items are qualitatively or quantitatively different from other items, or whether there are differences between NDI and Taxon items. In addition to the differences between NDI and Taxon items, the characteristics of each item group are also unclear. The DES have long been studied using CTT; however, CTT does not separate item characteristics from participant characteristics. By using item response theory (IRT), these can be separated. Saggino et al. [33] examined psychometric properties using the Partial Credit Model (PCM), an extension of the Rasch model, which is a type of item response theory. However, the method that Saggino et al. [33] used does not calculate the discriminative power of each item, hence the discriminative power of each item is unknown. Moreover, Saggino et al. [33] analyzed 25 items, excluding item 1, item 12, and item 21 out of the total 28 items. Since the DES-II is generally used with 28 items, it is necessary to clarify the psychometric properties of the three excluded items as well. Revealing the characteristics of each item would provide guidance on which items and which specific ratings should be given attention.

Secondly, there is little evidence regarding the longitudinal stability of the DES-II. The DES-II is a scale with temporal stability and has been used as an indicator of trait dissociation [26, 28, 29]. When measurements were spaced four weeks apart, ICC ranged from 0.80 to 0.93 [23, 27]. Test–retest reliability (i.e., correlation coefficients) was 0.94 [19], indicating consistent temporal stability. Putnam et al. [34] examined test–retest reliability over a one-year interval and reported a rank correlation coefficient of 0.78, although no similar reports were found.

However, longitudinal stability is expected to decrease as time progresses. A meta-analytic study [35] indicated that younger individuals have higher dissociative traits than older individuals, suggesting that dissociation may decrease with aging. Therefore, we could presume that temporal stability will decrease over time with yearly intervals. However, if the DES-II measures trait dissociation, a certain level of long-term stability should be observed. If long-term stability becomes evident, it can serve as a reference for whether changes in dissociative traits over time are due to the passage of time or due to effects of treatment and recovery. This is useful for evaluating long-term changes in dissociation.

### The present study

The present study has two aims. At first, we will examine the characteristics of the Japanese version DES-II. Saggino et al. [33] investigated psychometric properties using the Rasch model. However, three out of the 28 items were excluded from the analysis, and item difficulties and discriminative powers were not calculated. Hence, the present study will use a Grade Response Model (GRM) to examine the DES-II and investigate item difficulties and discriminative powers of each item. Secondly, we will examine the long-term stability of the Japanese version DES-II. While the DES has been reported to have high temporal stability (e.g., [23, 27]), the long-term stability at the annual level is not clear. Dissociation has been reported to decrease with aging [35]; therefore, we could presume that stability at the annual level will also decrease. To clarify this, we will investigate the long-term stability of the DES-II.

DES scores have long been computed based on the CTT. However, it is possible that the severity reflected by each items varies. By applying IRT to the DES-II, we could conduct a more detailed assessment that accounts for the severity levels of each rating for individual items. However, other than Saggino et al. [33], such studies are scarce internationally. Since the DES-II is used in the initial stage of dissociation screening, findings from community samples could provide valuable information for the early screening of dissociation.

## Method

### Participants

Participants of the present study were 1030 adults residing in Japan (515 females, 515 males) at Time 1 (T1), with a mean age of 44.64 (± 14.02). After excluding one participant with incomplete responses, data from 1029 participants (515 females, 514 males) with a mean age of 44.64 (± 14.02) was used for later analyses. At Time 2 (T2), out of the T1 participants, 210 individuals responded to the survey request (105 females, 105 males; *M*_*age*_ = 48.55 ± 13.74)); data from participants who responded to both surveys were included in the longitudinal analyses. The interval between T1 (July 19, 2017) and T2 (February 5, 2021) was approximately three and a half years.

No significant differences were observed in the DES-II scores or Taxon scores between participants who participated only at Time 1 and those who also participated at Time 2, and the effect sizes were small (*t* (304.65) = −0.65, *p* = 0.512, *d* = 0.05, 95% CI [−0.21, 0.10]; *t* (317.82) = −0.59, *p* = 0.55, *d* = 0.05, 95% CI [−0.20, 0.11]). Furthermore, there were no differences in demographic characteristics (gender: *χ*^2^ (1) ≒ 0.000, *p* ≒ 1.000; household income: *χ*^2^ (9) = 7.87, *p* = 0.546; marital status at T1: *χ*^2^ (1) = 0.222, *p* = 0.637; employment status at T1: *χ*^2^ (11) = 7.41, *p* = 0.765).

### Measures

We used the Japanese version of the Dissociative Experiences Scale II [19], which consists of 28 items; participants rated on an 11-point scale (0% to 100%). The internal consistencies were *α*_T1_ = 0.98 and *α*_T2_ = 0.96 in the present study. Several cut-off points have been proposed in previous studies [20–23]; we used the clear-cut-off point of 30, categorizing individuals scoring 30 or above as sub-clinical subjects (*N*_T1_ = 98 (9.52%), *N*_T2_ = 12 (5.71%)).

### Procedures

For T1, a large sample size was required to apply item response theory (IRT). On the other hand, for T2, a smaller sample size was sufficient since the purpose was to obtain intraclass correlations and rank correlations with T1 data. Considering that 9.5% of the participants at Time 1 were subclinical and the sample size required to detect a weak correlation coefficient (*r* = 0.20) was 193, a target sample size of 210 was set to account for potential invalid responses.

The survey targeted individuals registered as panels in a major online research company in Japan. All surveys were conducted online. Participants gave consent for participation over the internet. Procedures of the present study were approved by the Ethics committee of Graduate School of Education, Hiroshima University, and the Committee of Hiroshima Bunka Gakuen University School of Nursing (approval number: 2005). Before starting the survey, participants were informed that sensitive questions would be asked, and only those who agreed proceeded to the questionnaire. Furthermore, the online research company does not allow submission of responses with unanswered items, ensuring that all response data were complete. Therefore, there were no missing values in the collected dataset.

### Statistical analyses

In the present study, the significance level was set at *p* < 0.05. We calculated descriptive statistics including the mean, standard deviation, standard error, minimum and maximum values, kurtosis and skewness. To examine long-term stability of the DES-II, we computed ICC and Spearman’s rank correlation coefficient for the DES-II scores at T1 and T2. At T1, participants who provided only extreme responses (0% or 100%) to the DES-II were excluded from the analysis. At T2, three attention check items (e.g., “Please choose the rightmost”) were added to the DES-II to detect inattentive responses. Only data from participants who responded correctly to all check items were used for later analyses.

For the application of IRT, the DES-II must be unidimensional. However, as pointed out by Rubio et al. [36], cases where it is completely unidimensional are rare. Following the criteria outlined by Reckase [37] and Hambleton et al. [38], we considered that the DES-II was unidimensional if the variance explained by the first factor was above 20% and the eigenvalue of the first factor was more than five times that of the second factor.

IRT estimates individual ability values (*θ*) and the item parameter (item difficulties and discriminative powers) from the correctness of test items. Item discriminative power indicates the ability to distinguish between individuals with high and low abilities. Baker [39] provides criteria for evaluating discriminative power (a): *a* = 0: none; *a* = 0.01 ~ 0.34: very low; *a* = 0.35 ~ 0.64: low; *a* = 0.65 ~ 1.34: moderate; 1.35 ~ 1.69: high; *a* > 1.70: very high. On the other hand, item difficulty (*b*) represents the ability level (*θ*) required for a 50% correct response rate, with higher values indicating more difficult items.

Traditional IRT cannot be applied to psychological scales using the Likert scale, assuming multiple response options. When applying IRT to polytomous response data obtained from Likert scales, it is necessary to utilize models that assume graded responses, such as the Graded Response Model (GRM) [40, 41] or the Partial Credit Model (PCM) [42]. In the GRM, the difficulty parameters increase monotonically, meaning that the difficulty of a given item's rating is higher for larger ratings than for smaller ones, whereas the PCM does not assume monotonicity in the difficulty parameters, so there does not need to be consistency between the magnitude of the ratings and the item difficulty parameters [42]. Considering that prior research has shown that the GRM is suitable for analyzing Likert scales [43], we used the GRM for analysis. GRM is a model that extends the two-parameter logistic model to multi-rating data, estimating item difficulty and discriminative power. However, equality constraints are imposed on discriminative power for each step within an item. The GRM calculates an individual's ability parameter (*θ*) by considering the discrimination and difficulty parameters of scale items. In this study, this value represents the level of dissociative traits.

All analyses were performed using R (4.2.2). The "readxl" [44] and "readr" [45] packages were used for data loading, the "tidyverse" [46] package for data shaping and visualization, the "devEMF" [47] and "patchwork" [48] packages for figure output, the "psych" [49] package for calculating descriptive statistics, the "mirt" [50] package for estimating parameters based on item response theory, and the "irr" [51] package for calculation of the ICC.

## Results

### Demographic characteristics of study participants

Table 1 shows the characteristics of participants at T1, including gender, household income, marital status, presence of children, and employment status.

**Table 1:** Characteristics of participants

	*n*	%		*n*	%
Gender			child		
Female	514	49.95%	yes	567	55.10%
Male	515	50.05%	no	462	44.90%
Household income			employment status		
~ ¥2,000,000	80	7.77%	fulltime homemaker	207	20.12%
~ ¥4,000,000	213	20.70%	parttime job	146	14.19%
~ ¥6,000,000	205	19.92%	office others	134	13.02%
~ ¥8,000,000	125	12.15%	office worker	132	12.83%
~ ¥10,000,000	100	9.72%	office engineer	120	11.66%
~ ¥12,000,000	41	3.98%	unemployed	99	9.62%
~ ¥15,000,000	16	1.55%	self employed	56	5.44%
~ ¥20,000,000	9	0.87%	others	38	3.69%
¥20,000,000 ~	2	0.19%	student	32	3.11%
No answer	114	11.08%	public employee	31	3.01%
			free worker	22	2.14%
Current marital status			manager / executive	12	1.17%
Married	615	59.77%			
Divorced / never	414	40.23%			

Based on T1 data (*N* = 1029)

### Classical test theory

**Fig. 1:**
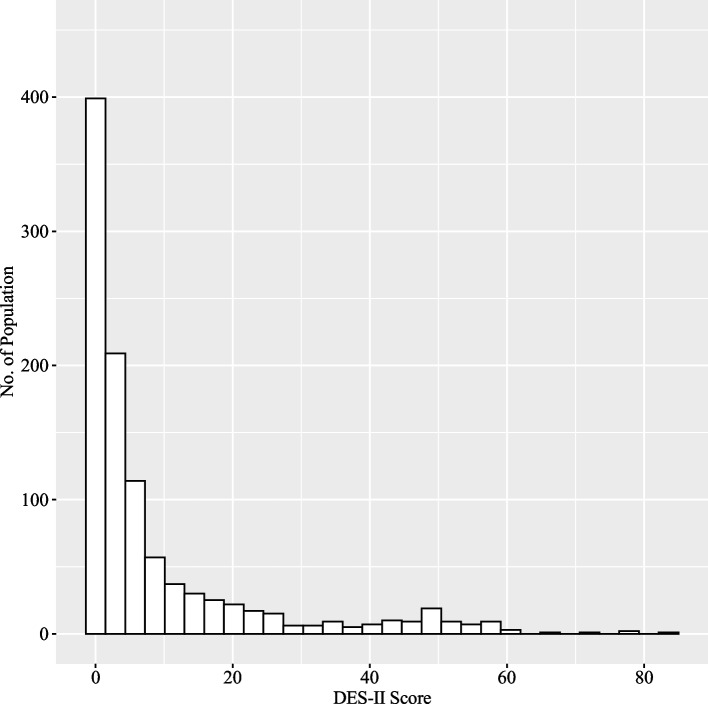
A histogram of the DES-II at Time 1 (*N* = 1029)

We calculated descriptive statistics for the DES-II. The distribution of the DES-II scores exhibited a skewed distribution with a L-shape (Fig. 1). The mean score of the DES-II was 9.04 (*SD* = 14.39, *SE* = 0.46), with a median of 2.86 (*25% quantile* = 0.71, *75% quantile* = 10.00). The minimum score was 0.00, and the maximum score was 83.57. The skewness was 2.47, which indicates right skewness, and the kurtosis was 5.56 indicating heavy tails in the distribution.

We calculated descriptive statistics for each item of the DES-II (Table 2). Skewness ranged from 1.55 (item 2) to 3.24 (item 4) indicating right-skewed distributions, and kurtosis ranged from 1.85 (item 2) to 10.22 (item 4), indicating heavy tails in the distribution. Finally, we computed item-total correlations for the DES-II, yielding *r*s = 0.56—0.78.

**Table 2:** Descriptive statistics of the DES-II

item	Taxon	*M*	*SD*	*SE*	*Mdn*	*Quantile*		*Min*	*Max*	*skew*	*kurtosis*	*r*_*it*_
						25%	75%					
item1	NDI	8.55	17.95	0.56	0.00	0.00	10.00	0	100	2.42	5.45	.68
item2	NDI	17.46	22.84	0.71	10.00	0.00	30.00	0	100	1.55	1.85	.78
item3	Taxon	7.20	17.06	0.53	0.00	0.00	0.00	0	100	2.78	7.53	.65
item4	NDI	5.17	15.11	0.47	0.00	0.00	0.00	0	100	3.24	10.22	.56
item5	Taxon	6.00	15.58	0.49	0.00	0.00	0.00	0	100	2.96	8.60	.59
item6	NDI	6.86	16.00	0.50	0.00	0.00	0.00	0	100	2.66	6.76	.59
item7	Taxon	7.23	17.43	0.54	0.00	0.00	0.00	0	100	2.73	7.08	.66
item8	Taxon	6.39	15.76	0.49	0.00	0.00	0.00	0	100	2.86	8.00	.60
item9	NDI	9.53	18.90	0.59	0.00	0.00	10.00	0	100	2.32	5.14	.64
item10	NDI	8.81	17.92	0.56	0.00	0.00	10.00	0	100	2.42	5.87	.68
item11	NDI	5.44	14.84	0.46	0.00	0.00	0.00	0	90	3.08	9.29	.58
item12	Taxon	8.67	17.91	0.56	0.00	0.00	10.00	0	100	2.32	4.94	.70
item13	Taxon	8.32	18.41	0.57	0.00	0.00	10.00	0	100	2.56	6.24	.67
item14	NDI	12.31	21.14	0.66	0.00	0.00	20.00	0	100	1.93	3.16	.69
item15	NDI	11.19	19.29	0.60	0.00	0.00	10.00	0	100	1.99	3.37	.73
item16	NDI	7.91	17.50	0.55	0.00	0.00	10.00	0	100	2.64	6.83	.67
item17	NDI	13.97	21.58	0.67	0.00	0.00	20.00	0	100	1.76	2.50	.71
item18	NDI	8.10	17.89	0.56	0.00	0.00	10.00	0	100	2.64	7.02	.68
item19	NDI	11.11	20.29	0.63	0.00	0.00	10.00	0	100	2.18	4.46	.64
item20	NDI	9.86	18.59	0.58	0.00	0.00	10.00	0	100	2.23	4.67	.70
item21	NDI	12.10	21.20	0.66	0.00	0.00	10.00	0	100	2.04	3.57	.65
item22	Taxon	9.12	18.99	0.59	0.00	0.00	10.00	0	100	2.34	5.00	.67
item23	NDI	10.90	19.52	0.61	0.00	0.00	10.00	0	100	2.00	3.50	.67
item24	NDI	10.54	19.24	0.60	0.00	0.00	10.00	0	100	2.08	3.77	.72
item25	NDI	8.45	17.03	0.53	0.00	0.00	10.00	0	100	2.42	5.80	.69
item26	NDI	7.84	16.59	0.52	0.00	0.00	10.00	0	90	2.40	5.07	.65
item27	Taxon	7.46	17.34	0.54	0.00	0.00	0.00	0	100	2.54	5.80	.64
item28	NDI	6.72	15.88	0.50	0.00	0.00	0.00	0	100	2.76	7.62	.65
Total	―	9.04	14.39	0.45	2.86	0.71	10.00	0	83.57	2.26	4.71	―
Taxon	―	7.55	14.86	0.46	0.00	0.00	7.50	0	82.5	2.47	5.56	―
NDI	―	9.64	14.43	0.45	3.50	0.50	11.50	0	84	2.14	4.24	―

*M* mean, *SD* standard deviation, *SE* standard error, *Mdn* median, *Min* minimam, *Max* maximum, *r*_*it*_ item-total correlation (spearman’s rank correlation)

### Evaluation of unidimensionality of the DES-II

Before conducting IRT analyses, we examined whether the DES-II could be assumed to be unidimensional. Initially, the variance explained by the first factor was 43%. This significantly exceeds the criterion set by Reckase [37] of 20% or more. Moreover, the eigenvalues for the first, second, and third factors were 17.96, 1.08, and 0.67, respectively. The ratio of the first eigenvalue to the second eigenvalue was 16.63, greatly surpassing the criterion established by Hambleton et al. [38] of 5 times or more. Therefore, we presumed that the DES-II was unidimensional.

### Item evaluation of the DES-II with IRT

#### Visualization of scale characteristics

**Fig. 2:**
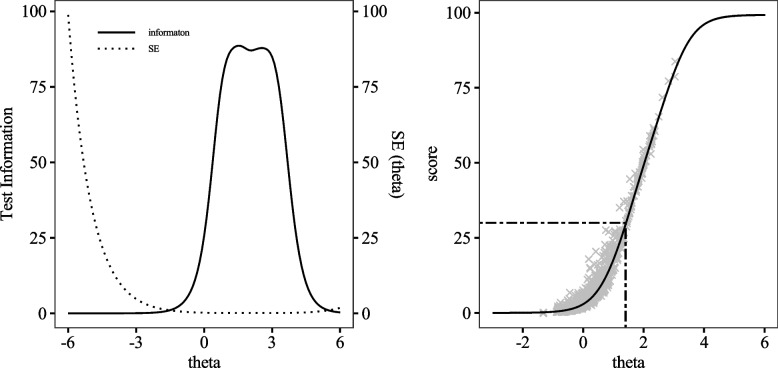
TIC (left) and TCC (right) for the DES-II. The dashed line in TCC is the cut-off point for the DES-II and the corresponding *θ*

We plotted the Test Characteristic Curve (TCC) and Test Information Curve (TIC) (Fig. 2). TCC is a curve that shows the correspondence between raw scores and the *θ* values. In the present study, it represents the relationship between the DES-II scores and *θ* values, allowing the *θ* value corresponding to the cut-off point to be identified. The TCC indicated that the cut-off point for the DES-II (total score = 30) corresponds to *θ* = 1.40. TIC is a curve that illustrates the relationship between test information and *θ* values. In the present study, it identifies the range of *θ* levels that the DES-II can measure with high precision. The TIC showed maximum information at *θ* = 1.50, suggesting that the DES-II exhibits adequate measurement precision in the range of *θ* = 1.00 to 3.00.

**Fig. 3:**
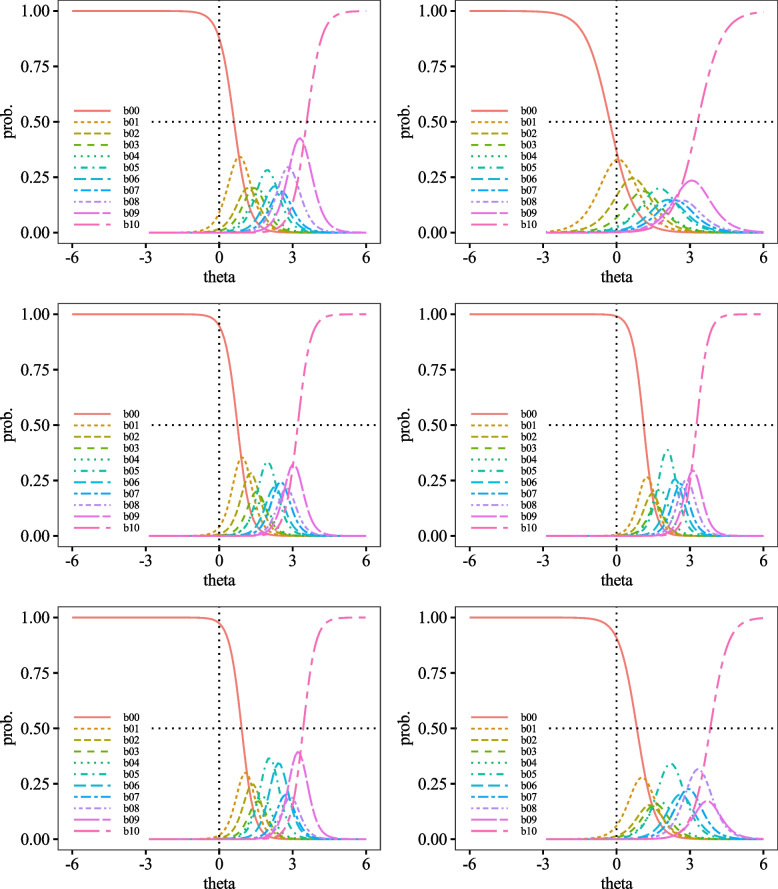
Category Probability Curves for item1 (top left), item2 (top right), item3 (middle left), item4 (middle right), item5 (bottom left), and item6 (bottom right)

**Fig. 4:**
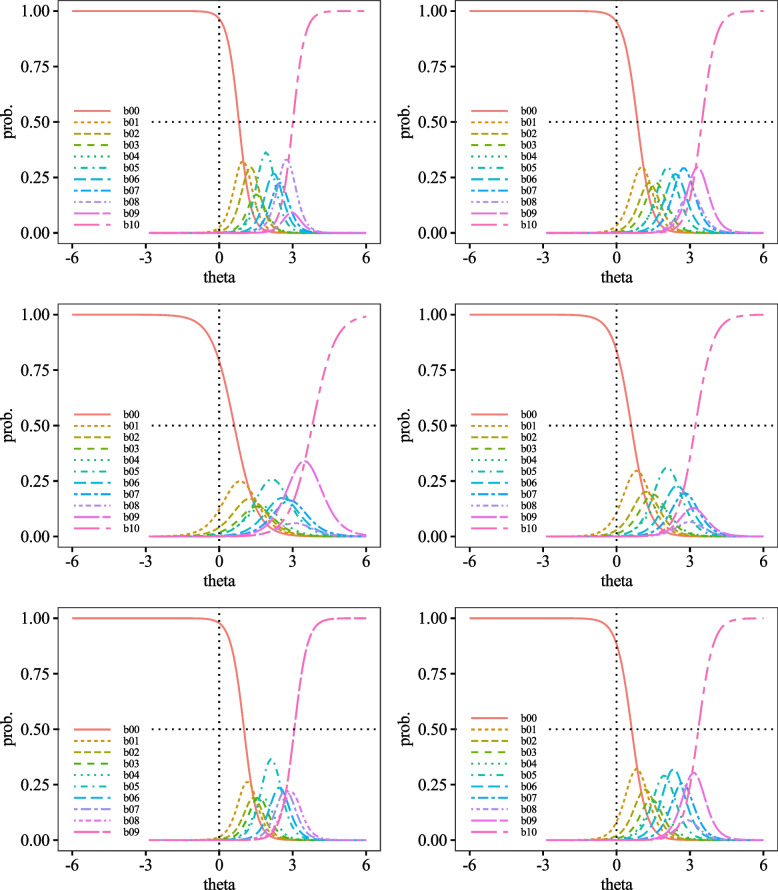
Category Probability Curves for item7 (top left), item8 (top right), item9 (middle left), item10 (middle right), item11 (bottom left), and item12 (bottom right)

**Fig. 5:**
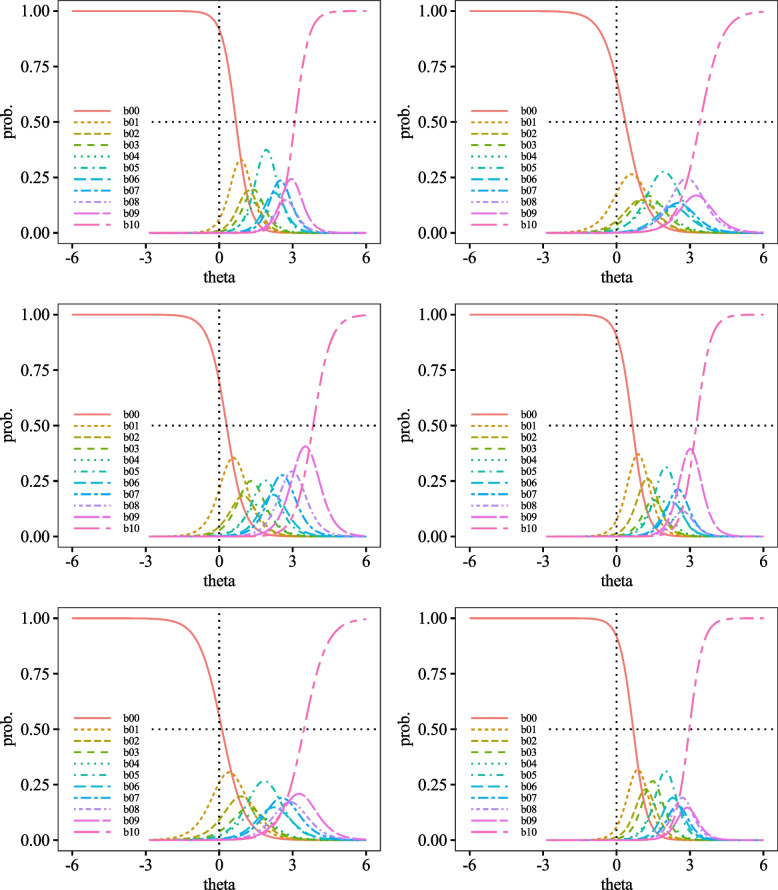
Category Probability Curves for item13 (top left), item14 (top right), item15 (middle left), item16 (middle right), item17 (bottom left), and item18 (bottom right)

**Fig. 6:**
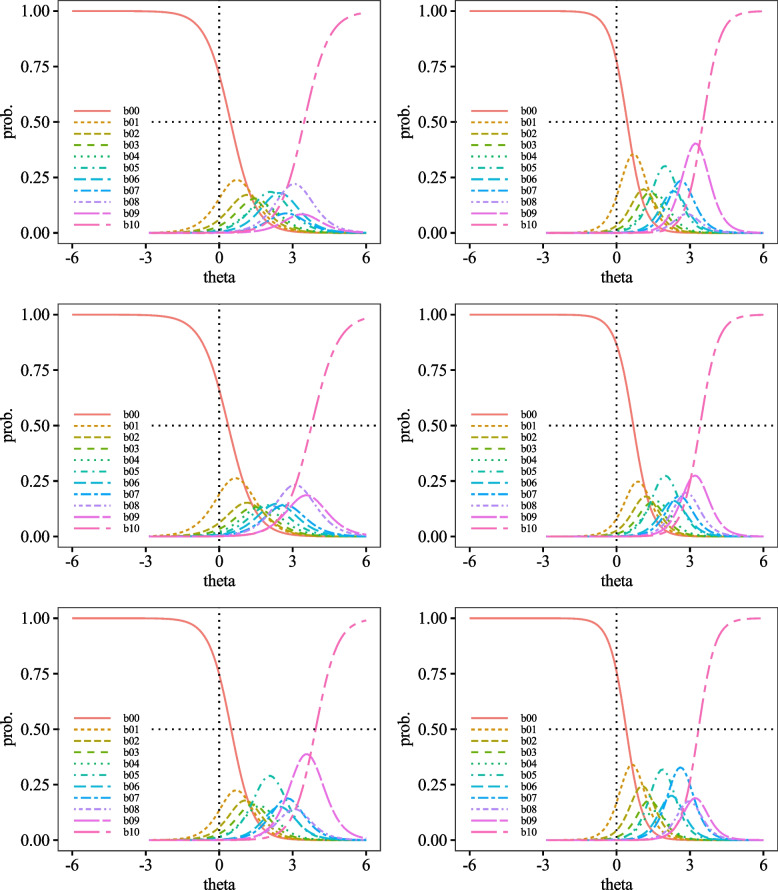
Category Probability Curves for item19 (top left), item20 (top right), item21 (middle left), item22 (middle right), item23 (bottom left), and item24 (bottom right)

**Fig. 7:**
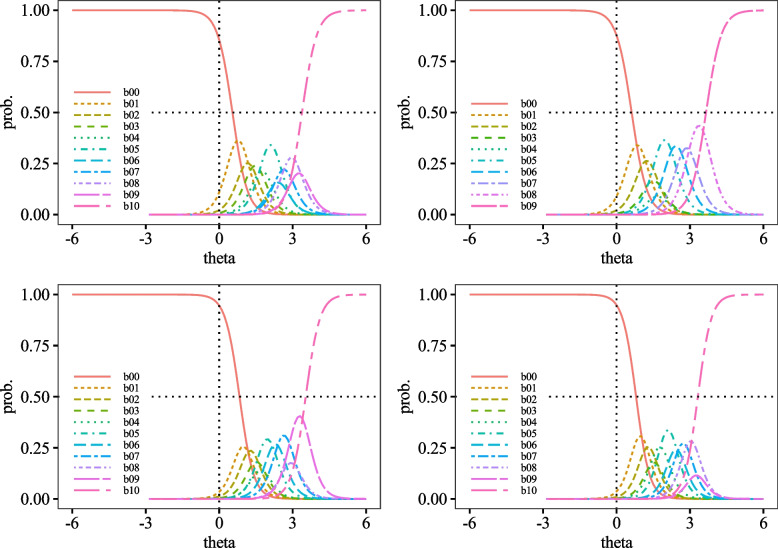
Category Probability Curves for item25 (top left), item26 (top right), item27 (bottom left), and item28 (bottom right)

Category Response Curves (CRCs) are visual representations of the discrimination and difficulty of ratings for each item. Discrimination corresponds to the slope of the curve, while difficulty corresponds to the position of the curve. The closer the curve is to the left, the lower the difficulty of the rating, and vice versa. CRCs for each item were positioned to the right of *θ* = 0.00 (Figs. 3, 4, 5, 6 and 7).

### Item parameters

Table 3 shows the item parameters. The estimated difficulty values ranged from *b*_1_ = −0.272 to 1.108 (*M* = 0.602, *SD* = 0.281). The standard deviation of difficulty was small at all stages, indicating the absence of notably difficult or easy items. In reference to Baker's criteria [39], the discriminability of all items was very high (*a* = 1.828 to 4.260, *M* = 3.047, *SD* = 0.704); thus, all items in the DES-II were deemed useful in discriminating the degree of dissociative characteristics.

**Table 3:** Item parameters estimated by GRM

item	Taxon	*a*	*b*_1_	*b*_2_	*b*_3_	*b*_4_	*b*_5_	*b*_6_	*b*_7_	*b*_8_	*b*_9_	*b*_10_	*S-χ*^*2*^	*df*	*p*
item1	NDI	3.25	0.60	1.05	1.30	1.55	1.78	2.14	2.40	2.64	3.01	3.57	116.90	100	.119
item2	NDI	2.01	−0.27	0.41	0.91	1.29	1.55	1.95	2.25	2.54	2.84	3.31	145.63	133	.214
item3	Taxon	3.89	0.74	1.13	1.43	1.63	1.79	2.15	2.38	2.64	2.86	3.21	104.72	83	.054
item4	NDI	4.26	1.11	1.37	1.55	1.67	1.88	2.26	2.51	2.73	2.98	3.27	95.60	56	.001
item5	Taxon	3.96	0.92	1.23	1.49	1.66	1.86	2.25	2.61	2.82	3.01	3.43	113.61	73	.002
item6	NDI	2.75	0.83	1.25	1.47	1.71	1.95	2.47	2.76	3.10	3.58	3.83	105.73	88	.096
item7	Taxon	4.13	0.81	1.13	1.43	1.60	1.72	2.09	2.36	2.58	2.92	3.01	117.82	83	.007
item8	Taxon	3.49	0.85	1.21	1.47	1.74	1.91	2.26	2.57	2.92	3.14	3.49	107.36	81	.027
item9	NDI	2.16	0.62	1.09	1.41	1.66	1.91	2.40	2.73	3.03	3.15	3.80	119.99	105	.150
item10	NDI	2.73	0.60	1.05	1.35	1.63	1.83	2.30	2.64	2.93	3.03	3.22	109.75	104	.331
item11	NDI	3.75	1.01	1.30	1.51	1.72	1.92	2.33	2.59	2.83	3.07	―^a^	68.77	63	.288
item12	Taxon	3.28	0.62	1.03	1.31	1.54	1.76	2.12	2.53	2.85	2.96	3.34	112.92	101	.196
item13	Taxon	3.50	0.68	1.08	1.30	1.52	1.69	2.15	2.36	2.63	2.80	3.09	84.68	88	.581
item14	NDI	2.22	0.36	0.85	1.12	1.43	1.67	2.19	2.40	2.65	3.10	3.41	146.28	128	.129
item15	NDI	2.75	0.30	0.84	1.11	1.49	1.70	2.07	2.35	2.77	3.21	3.83	116.83	115	.435
item16	NDI	3.45	0.65	1.11	1.42	1.61	1.82	2.20	2.38	2.64	2.77	3.25	101.64	91	.209
item17	NDI	2.13	0.11	0.70	1.08	1.35	1.58	2.09	2.37	2.74	3.06	3.46	123.01	131	.678
item18	NDI	3.55	0.68	1.05	1.32	1.63	1.82	2.19	2.41	2.59	2.81	2.97	109.65	89	.068
item19	NDI	1.96	0.47	0.96	1.32	1.64	1.90	2.28	2.66	2.84	3.30	3.47	125.33	117	.282
item20	NDI	2.83	0.44	0.96	1.24	1.51	1.76	2.20	2.47	2.80	2.93	3.53	101.12	107	.642
item21	NDI	1.83	0.37	0.96	1.29	1.59	1.89	2.20	2.52	2.84	3.36	3.77	132.64	122	.240
item22	Taxon	2.73	0.69	1.06	1.32	1.55	1.78	2.20	2.44	2.71	3.00	3.42	120.92	99	.067
item23	NDI	2.22	0.49	0.90	1.22	1.52	1.79	2.33	2.60	2.95	3.20	3.94	115.49	113	.417
item24	NDI	2.93	0.39	0.88	1.22	1.47	1.65	2.10	2.38	2.84	3.09	3.35	122.05	110	.203
item25	NDI	3.28	0.54	1.01	1.32	1.62	1.88	2.31	2.51	2.78	3.13	3.38	112.08	92	.076
item26	NDI	3.19	0.62	1.06	1.40	1.61	1.72	2.21	2.65	3.07	3.66	―^a^	138.61	97	.004
item27	Taxon	3.45	0.83	1.13	1.41	1.62	1.79	2.14	2.46	2.83	3.03	3.53	100.84	84	.102
item28	NDI	3.65	0.80	1.15	1.43	1.63	1.92	2.30	2.57	2.88	3.20	3.32	77.55	84	.677
*M*	Total	3.05	0.60	1.03	1.33	1.58	1.79	2.21	2.49	2.79	3.08	3.43			
*SD*	Total	0.70	0.28	0.19	0.14	0.10	0.10	0.11	0.13	0.15	0.21	0.25			
*Min*	Total	1.83	−0.27	0.41	0.91	1.29	1.55	1.95	2.25	2.54	2.77	2.97			
*Max*	Total	4.26	1.11	1.37	1.55	1.74	1.95	2.47	2.76	3.10	3.66	3.94			
*SE*	Total	0.13	0.05	0.04	0.03	0.02	0.02	0.02	0.02	0.03	0.04	0.05			
*M*	NDI	2.84	0.54	1.00	1.30	1.57	1.80	2.23	2.51	2.81	3.12	3.48			
*SD*	NDI	0.69	0.30	0.21	0.16	0.11	0.12	0.12	0.14	0.16	0.23	0.26			
*Min*	NDI	1.83	−0.27	0.41	0.91	1.29	1.55	1.95	2.25	2.54	2.77	2.97			
*Max*	NDI	4.26	1.11	1.37	1.55	1.72	1.95	2.47	2.76	3.10	3.66	3.94			
*SE*	NDI	0.15	0.07	0.05	0.04	0.03	0.03	0.03	0.03	0.04	0.05	0.06			
*M*	Taxon	3.55	0.77	1.12	1.39	1.61	1.79	2.17	2.46	2.75	2.97	3.31			
*SD*	Taxon	0.44	0.10	0.07	0.07	0.07	0.07	0.06	0.10	0.12	0.10	0.19			
*Min*	Taxon	2.73	0.62	1.03	1.30	1.52	1.69	2.09	2.36	2.58	2.80	3.01			
*Max*	Taxon	4.13	0.92	1.23	1.49	1.74	1.91	2.26	2.61	2.92	3.14	3.53			
*SE*	Taxon	0.16	0.04	0.02	0.03	0.03	0.02	0.02	0.03	0.04	0.04	0.07			

*NDI* item of Normal Dissociation Index, *Taxon* item of DES-T, *b* item threshold parameters, *a* item discrimination parameter, *M* mean, *SD* standard deviation, *Min* minimum, *Max* maximum, *SE* standard error

^a^It was not calculated because there were no respondents who answered “100%”

### Goodness of fit

The model fit was excellent, with *CFI* = 0.994, *TLI* = 0.992, *RMSEA* = 0.022 (95% CI [0.014, 0.029]), *AIC* = 48418.81, and *BIC* = 49929.33. While the item fit was generally adequate, the fit tests for items 4, 5, 7, 8, and 26 were significant (*S-χ*^2^ = 95.60, *p* = 0.001; *S-χ*^2^ = 113.61, *p* = 0.002; *S-χ*^2^ = 117.82, *p* = 0.007; *S-χ*^2^ = 107.36, *p* = 0.027; *S-χ*^2^ = 138.60, *p* = 0.004).

### Longitudinal stability of the DES-II

**Fig. 8:**
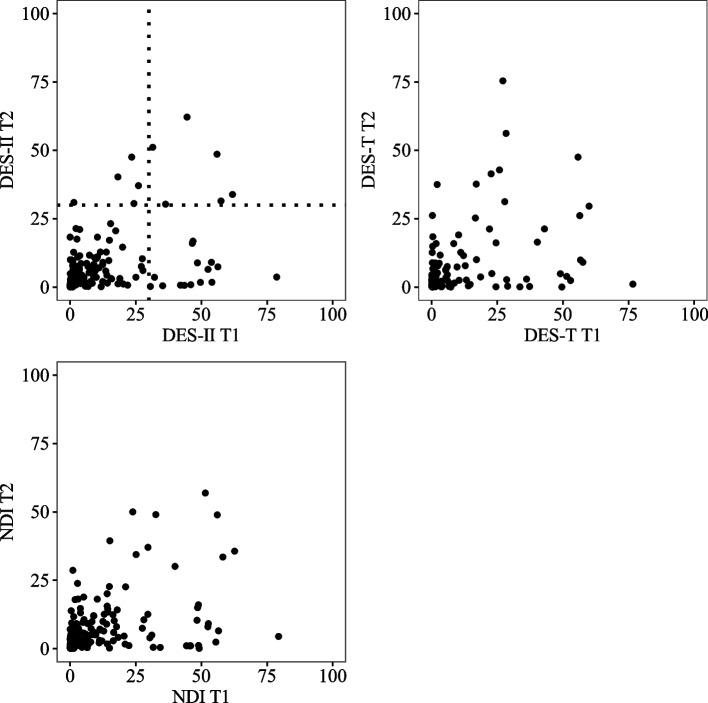
Scatter plots of the DES-II scores at T1 and T2. The dotted line represents the DES-II cut-off point (DES-II = 30)

To visualize the relationship between the DES-II scores at T1 and T2, we created a scatterplot (Fig. 8). To examine the temporal stability of the DES-II scores, we calculated the *ICC* between T1 and T2. Results showed a significant moderate within-class correlation coefficient (*ICC* = 0.51, *F* (209, 167) = 2.11, *p* < 0.001, 95% CI [0.35, 0.63]). Spearman's rank correlation coefficient between T1 and T2 was also significant, showing moderate correlation (*r* = 0.48 (95% CI [0.36, 0.57], *p* < 0.001).

## Discussion

In the present study, we assessed the psychometric properties of the DES-II using the IRT analysis. Our results suggested that the DES-II is a suitable instrument for measuring strong dissociative traits, and that the cut-off points lie within an acceptable range of measurement accuracy. The long-term stability over an interval of approximately 3.5 years was also adequate. All DES-II items were highly discriminative and contributed to the measurement of dissociative properties. The difficulty level was generally high, with 27 of the 28 items requiring *θ* > 0, even when answering “10%” on the DES-II. However, no consistent trend in difficulty was detected. In addition, there were several NDIs with higher discrimination than the Taxon items; no psychometric features distinguishing the Taxon items from the NDIs were identified.

Results from the GRM assuming a single-factor structure demonstrated that the DES-II exhibits excellent properties. The TIC indicated that the DES-II is most accurately measured within *θ* = 1.00 to 3.00, suggesting that the DES-II is suitable for measuring strong dissociative traits. According to the TCC, the cut-off point of the DES-II (DES-II = 30 [21, 22]) corresponds to *θ* = 1.40. This is close to *θ* where the test information is maximized, supporting the cut-off points proposed by Bernstein et al. [21] and Zingrone & Alvarado [22]. Furthermore, the criteria proposed by Steinberg et al. [20] (total score is 15–20) corresponds to *θ* = 0.80–1.10, and Frischholz et al.’s criteria (total score is 45–55) [23] corresponds to *θ* = 1.80–2.00. Based on our findings, Steinberg et al.’s criteria [20] set the cut-off point in a range where the measurement accuracy of the DES-II is not high; hence, the cut-off point (15–20) that they proposed may not be suitable for screening individuals with strong dissociative traits.

The performance of each item of the DES-II was also adequate, suggesting that the scale comprises items suitable for measuring strong dissociative traits. First, regarding difficulty, the variance from *b*_1_ to *b*_10_ was almost uniform, indicating that the DES-II does not include excessively difficult or easy items. The CRCs were generally positioned to the right. Other than curve *b*_0_ (corresponding to the response "0%"), none of the peaks of the CRCs were located to the left of *θ* = 0.00. Additionally, most CRCs for choices *b*_1_ to *b*_9_, corresponding to 10% to 90%, overlapped with some curves positioned inside the arcs drawn by adjacent CRCs. Since CRCs’ vertical axis represents response probabilities, choices with significant overlaps are less likely to be selected. For instance, item 9 had *b*_1_ positioned inside *b*_0_. Since the peak of *b*_0_ was higher than b00’s, *b*_0_ was more likely to be chosen, making *b*_1_ less likely to be selected. Moreover, the discrimination parameters had small variances, with all *a* > 1.00. No items had 0.00 < *a* < 1.00, indicating that there were no items that contributed poorly to measuring dissociative characteristics. As there were no items with *a* < 0.00, the DES-II likely does not include items contradictory to measuring dissociative properties. Hence, it can be said that all items in the DES-II are suitable for measuring dissociation.

Distinct psychometric properties between NDI and Taxon were not identified. Taxon items exhibited lower variance in discrimination (*SD*_Taxon_ = 0.44 vs. *SD*_NID_ = 0.69), indicating that they were relatively homogeneous. Similarly, item difficulties were also homogeneous, suggesting that DES-T is measured by items with homogeneous dissociative properties than NDI. However, we could not detect differences in item parameters between Taxon items and some NDI items, such as item 3 (Taxon) and item 16 (NDI). Hence, it is unclear whether there are distinct psychometric characteristics between NDI and Taxon items.

Considering results from Saggino et al. [33], response patterns for the DES-II may vary depending on the participants' country of origin. Based on the Rasch model, Saggino et al. [33] excluded several items (item1, 12, and 21), but these items showed a good item fit in the present study. Participants included in our study were approximately five years older and had a larger age variance compared to Saggino et al. [33]. In addition, there were differences in descriptive statistics for each item, with items 1, 2, 14, 23, and 24 having higher means in Saggino et al. [33]. Therefore, besides differences in the applied models, discrepancies between results of the present study and Saggino et al. [33] may stem from differences in the original response patterns; differences may lie in the participants’ demographics.

Saggino et al. [33] included Italian participants, including community residents and incarcerated individuals, while our study comprised a sample of Japanese community members only. Regarding the comparison of DES-II scores between Eastern and Western populations, Carlson & Rosser-Hogan [52] reported that individuals exposed to trauma in Eastern and Western cultures had comparable the DES-II scores. Ross et al. [53] compared dissociative symptoms between Canada and China: while there was a significant difference in DES-II scores, no significant difference was observed in Taxon scores. Dunn et al. [54] compared the DES-II scores between African Americans and Caucasians, reporting significantly higher scores among the former. Kleindorfer [55] compared Chinese and Japanese populations, suggesting that response patterns vary due to socio-cultural factors. According to Chen et al. [56], although there are differences in response tendencies to questionnaires between Eastern and Western populations, these differences do not significantly affect cross-cultural comparisons; hence, we could assume that pathological dissociation shows no substantial differences between Eastern and Western populations, but everyday dissociative experiences may differ. Such differences are likely to influence response patterns to the DES-II. Our results suggest the possibility of cultural differences in item parameters for DES-II, indicating the need for further research using the DES-II in different countries and regions. However, since there are various countries and regions in the East (or West) with diverse cultures, researchers should be cautious with over-simplification.

The second aim of this study was to evaluate the long-term stability of the DES-II. Research on temporal stability was limited, especially concerning long-term stability. In previous studies, temporal stability was assessed over several weeks, showing high stability (*ICC*s = 0.80—0.93 [23, 27]). The retest reliability of the Japanese version was *r* = 0.94 [19], confirming high temporal stability. Regarding long-term stability, Putnam et al. [34] set a one-year interval, reporting a rank correlation coefficient of *r* = 0.78. The DES-II is commonly used for assessing dissociation, and insights into long-term temporal stability are valuable for investigating the effects of interventions over extended periods. Our results suggest that dissociative properties measured by the DES-II change over the years. With an interval of approximately 3.5 years, our study revealed lower stability than previous research (*ICC* = 0.51 (*F* (209, 167) = 2.11, *p* < 0.001, 95% CI [0.35, 0.63]), *r* = 0.48 (95% CI [0.36, 0.57], *p* < 0.001)). Tanabe [19] reported *r* = 0.94 with a four-week interval, and Putnam et al. [34] found *r* = 0.78 with a one-year interval, while our study showed 0.48 over three and a half years. Stability is gradually decreasing, indicating that dissociative properties change over the years. The possibility of a decline in dissociative properties with age has been previously suggested; Van IJzendoorn & Schuengel [35] conducted a meta-analysis showing that younger individuals have higher DES-II scores than older individuals. Our results are in line with their meta-analytic study, indicating that degrees of dissociative experiences may be affected by aging.

### Clinical implications and limitations

In clinical practice, attention should be paid to item scores in the DES-II. Screening and evaluation have traditionally been based on total scores; however, difficulty of the DES-II varies across items. For example, a response of 10% to item 4 and 20% to item 16 suggests the same level of dissociative traits. Notably, item 4 has relatively high discrimination and difficulty for b1 among all items, making it particularly noteworthy in the initial assessment of dissociation. If a respondent answers 10% or more for item 4, they may be experiencing dissociation comparable to answering 20%–30% for other items. Because this item has high difficulty even at low ratings, a rating as low as 20% may indicate significant dissociation. In contrast, items 2 and 7 have relatively low difficulty. Due to their high discrimination and wide range of difficulty, these items can capture a broad range of severity, from mild to severe, making them valuable for initial assessments in community samples.

When scorings are based on traditional CTT, a uniform response of 30% to all items would meet the cut-off point, focusing only on whether a respondent exceeds or falls below the cut-off. However, evaluating all items with the same weight may lead to overestimation of dissociation. According to the findings of the present study, items 3–9, 11, 16, and 26–28 (12 items in total) reach θ = 1.40 (the *θ* value corresponding to the cut-off point) at a rating of 30%. Items 1, 10, 12–15, and 18–25 require a rating of 40% to correspond to the cut-off level of severity, while items 2 and 17 only reach the cut-off severity level at a rating of 50%. Thus, setting cut-off points between 30 and 50% for individual items allows for more accurate assessments. Specifically, items requiring detailed screening at ratings of 30% or higher (items 3–9, 11, 16, and 26–28), 40% or higher (items 1, 10, 12–15, and 18–25), and 50% or higher (items 2 and 17) should be organized. If inconsistencies arise in the aggregated results, priority should be given to items with high discrimination for assessment. These findings suggest that item scores are as important as scale scores. Future assessments should incorporate the difficulty of ratings and the discrimination of items to achieve more precise evaluations.

Furthermore, when using the DES-II to evaluate treatment effects over several years, it is crucial to consider the decline in the DES-II scores over time. Our study supports the age-related decline in dissociative properties suggested by Van IJzendoorn & Schuengel [35] based on longitudinal analyses. Our results indicate that the DES-II scores show reduced temporal stability over several years. Hence, when evaluating changes in the DES-II scores over several years, careful consideration is needed to discern whether the change is due to treatment or the passage of time.

While our study provides valuable insights, there are several areas for improvement. Firstly, there are several limitations related to sampling procedures. The present study did not include a clinical sample; our findings are based on data from the general population. Frequencies of dissociative experiences differ between the general population and clinical groups, and previous studies suggest that dissociative experiences in the general population may decrease with age [57]. While Calciu et al. [57] was cross-sectional, it is possible that dissociative experiences in the general population are more prone to change than in clinical populations. Future research should include both general and clinical samples to compare response patterns to the DES-II and the extent of time-related changes. We also did not ask participants about life events that they experienced between T1 and T2. Various life events can occur over three and a half years, including marriage, employment, divorce, bereavement, and disaster experiences, all of which may be associated with dissociation. Therefore, to examine changes in dissociative properties solely due to the passage of time, statistically controlling for effects of various life events is necessary. Hence, future studies should re-examine long-term stability while controlling for these factors. Additionally, no efforts were made to encourage sustained participation in the study. It may have been necessary to implement strategies to encourage more participation in the T2 survey (e.g., offering higher incentives for cooperation in T2). Future studies may consider implementing strategies to enhance participant motivation for continued involvement in the study.

Secondly, there are methodological limitations related to measurement. The findings of this study are based on self-reported measures. Consequently, the relationship between *θ* values derived from IRT and the actual severity of dissociative symptoms has not been examined. Future research investigating the psychometric properties of the DES-II should examine its correspondence with external criteria, such as structured interviews or psychiatric diagnoses, and further explore the relationship between *θ* values and symptoms. Moreover, the measures regarding inattentive or dishonest responses at T1 were not entirely sufficient. Ideally, the same attention check items used at T2 should have been applied at T1.

Lastly, our study did not examine cultural influences. Future research should confirm whether our results stand for individuals from other countries or cultural backgrounds as well. As mentioned earlier, response patterns for the DES-II may vary due to socio-cultural factors [55]. However, there are few studies examining item response patterns for the DES-II, both in Western and Eastern contexts, and specific cultural differences remain unclear. The lack of knowledge about item response patterns makes it challenging to assess dissociative experiences in a manner appropriate to each culture. Therefore, future research should collect data not only from Japanese participants but also from Western participants to investigate cultural differences in item responses and clarify the impact of culture on dissociative experiences.

## Conclusion

The DES-II is recommended for use in initial screening for dissociation and is frequently used in clinical and research settings to assess dissociative symptoms; the DES-II assessment uses a mean score or cut-off point, and the presence of dissociation is suspected when scores exceed 30. However, the psychometric properties underlying the DES-II ―the characteristics of each item, the differences between the Taxon and NDI items, and the long-term stability of the DES-II scores― were not clear. Previous studies have analyzed the DES-II based on CTT and have applied IRT to only a few cases, and we found only one case of application of IRT using the Rasch model. The psychometric features of the DES-II would allow for detailed assessment based on responses to the items. Our results suggest that the DES-II cut-off point is reasonable from an IRT perspective and has excellent features for clinical use. And when there is a low-scoring response to the DES-II, an assessment that focuses on the response to specific items is necessary. Furthermore, the different levels of difficulty for each item suggest that assessment focusing on responses to each item is better than assessment based on mean scores or cut-off points.

## Data Availability

The datasets analysed during the current study are not publicly available due to the condition that research participants consented under the agreement that the raw data would not be made public but are available from the corresponding author on reasonable request.

## Abbreviations

DES: Dissociative Experiences Scale
IRT: Item Response Theory
DDIS: Dissociative Disorder Interview Schedule
SCID-D-R: Structured Clinical Interview for DSM-IV Dissociative Disorders-Revised
DSM: Diagnostic and Statistical Manual of Mental Disorders
MID: Multidimensional Inventory of Dissociation
NDI: Normal Dissociation Index
DES-T: DES-Taxon
ICC: Intraclass Correlation Coefficient
PCM: Partial Credit Model
GRM: Grade Response Model
TCC: Test Characteristic Curve
TIC: Test Information Curve
CRC: Category Probability Curve
